# Major Complication Caused by Inguinal Hernia Recurrence After Percutaneous Internal Ring Suturing Procedure in a Patient With Loeys-Dietz Syndrome: A Case Report

**DOI:** 10.7759/cureus.61449

**Published:** 2024-05-31

**Authors:** Aleksandra I Sadecka, Marek Wolski

**Affiliations:** 1 Department of Pediatric Surgery, Medical University of Warsaw, Warsaw, POL

**Keywords:** loyes-diaz syndrome, percutaneous internal ring suturing, laparoscopic treatment, pirs, indirect inguinal hernia

## Abstract

Inguinal hernia repair is one of the most frequently performed procedures in pediatric surgery. Treatment methods include classical open repair and laparoscopic approach. In this report we analyze a case of a 14-month-old boy with Loeys-Dietz syndrome treated for an inguinal hernia with laparoscopic percutaneous internal ring suturing (PIRS). Two weeks post-operatively the patient was diagnosed with a recurrence of the hernia complicated by an intestine strangulation. As a re-operation of the hernia, the Lichtenstein method was applied successfully. We analyzed the literature to determine the safety and possible contradictions of the PIRS procedure, with particular emphasis on patients with comorbidities such as connective tissue disorders. We conclude that in the PIRS procedure, despite its safety, feasibility and low complication rate in healthy patients, too few studies were made to draw similar conclusions for patients with comorbidities such as connective tissue disorders.

## Introduction

Inguinal hernia is a very common problem in the pediatric population. Nowadays the surgical approach to this disease offers traditional open repairs and a variety of laparoscopic techniques. In our institution the percutaneous internal ring suturing (PIRS) method is widely used. Literature provides clinicians with a wide range of data concerning long-term results, surgical techniques, and safety assessments of the PIRS procedure, but the number of publications presenting complications and possible contraindications is limited. For example, hydrocele or intestine incarceration is considered a contradiction to apply a laparoscopic approach in some studies and an indication in others [1,2].

Loeys-Dietz syndrome is an autosomal dominant heterogenous connective tissue disorder that mainly affects cardiovascular, musculoskeletal, and neurological systems and is characterized by a high incidence of inguinal hernia [3]. 

## Case presentation

**Figure 1:**
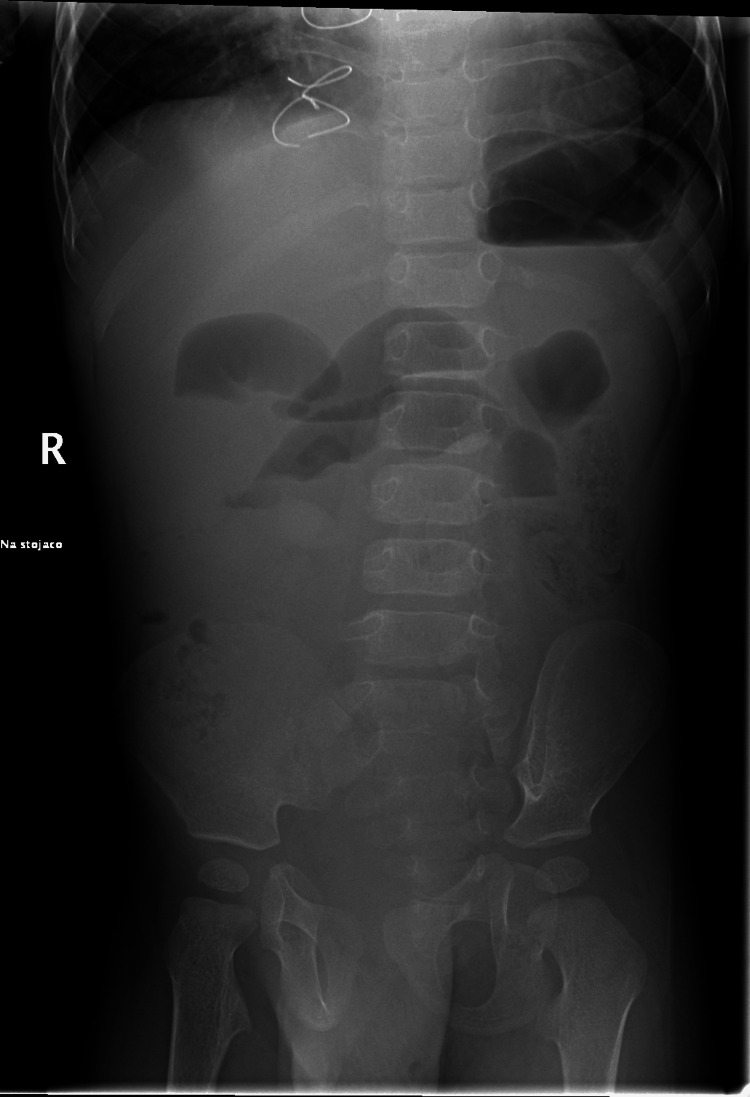
Abdominal X-ray showing dilated loops with fluid levels confirming intestinal obstruction

A 14-month-old boy with Loeys-Dietz type 3 syndrome was admitted to the hospital for an elective operation of the left-sided inguinal hernia. The patient was qualified for the laparoscopic percutaneous internal ring suturing in order to perform the shortest procedure, and at the same time to exclude or repair contralateral hernia. The PIRS procedure was successful, the contralateral patent processus vaginalis was excluded, the time under anesthesia was short, no intra-operative complications were noted. Two weeks later the patient was readmitted to the hospital due to recurrence of the hernia with a bowel incarceration. The surgeon on duty attempted sedation and manual reduction of the hernia, which seemed successful. The next day the patient presented symptoms of an intestinal obstruction. An abdominal X-ray confirmed this diagnosis (Figure 1).

**Figure 2:**
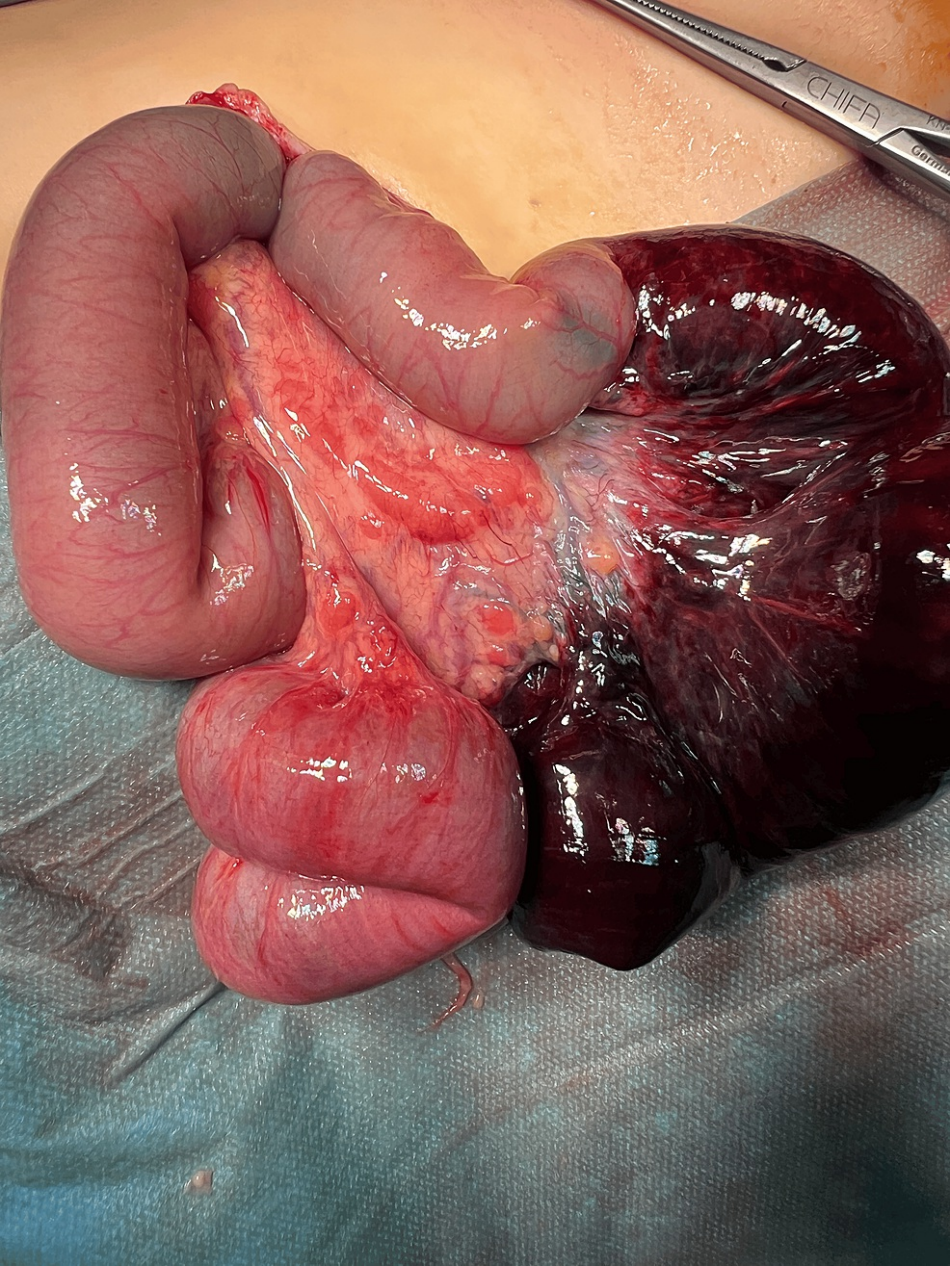
Intestinal obstruction caused by strangulation of the small intestine in the inguinal canal Ischemic part of the intestine before resection

**Figure 3:**
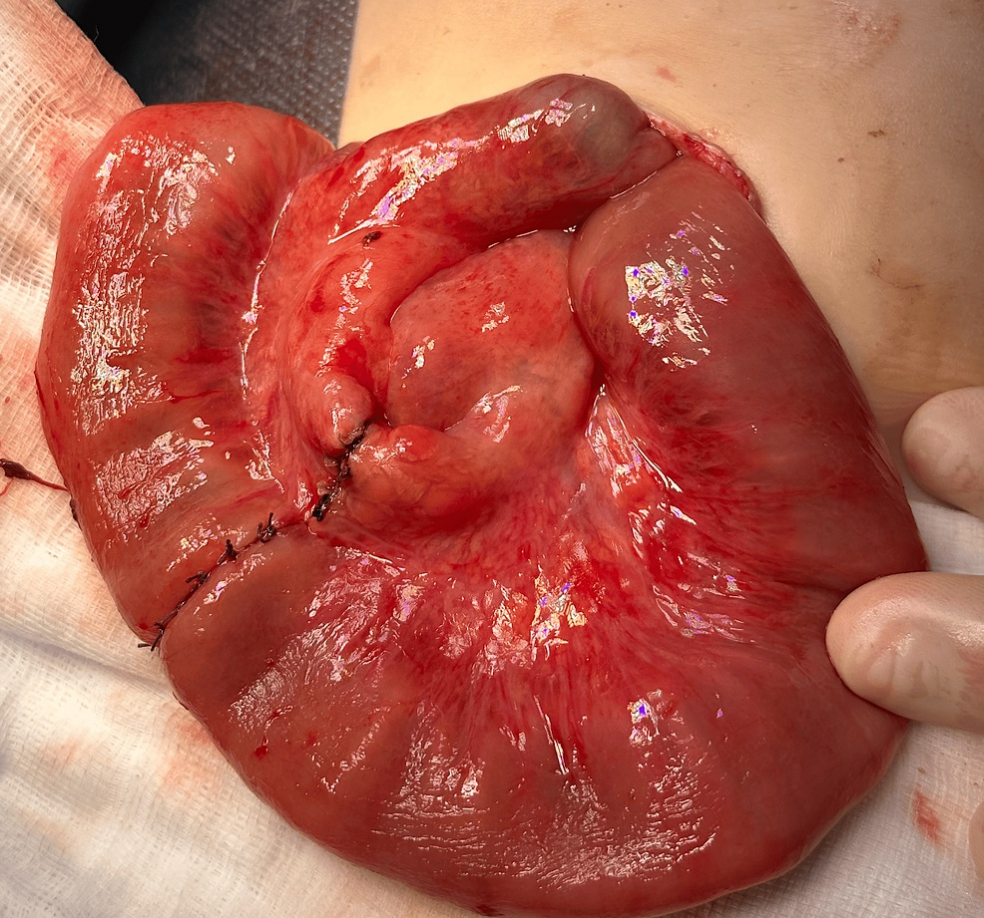
End-to-end anastomosis of the small intestine after resection of the ischemic part

The patient was qualified for an immediate laparoscopy which revealed a strangulation of the small intestine in the inguinal canal. Assessment of the inguinal ring area showed that the PIRS suture on the internal ring remained intact, but pulled up to the upper part of the canal as the bowel loop protruded into the canal below it pushing out another fragment of the flexible peritoneum. Mini-laparotomy was performed. The ischemic intestine was resected and an anastomosis was performed (Figures 2, 3).

**Figure 4:**
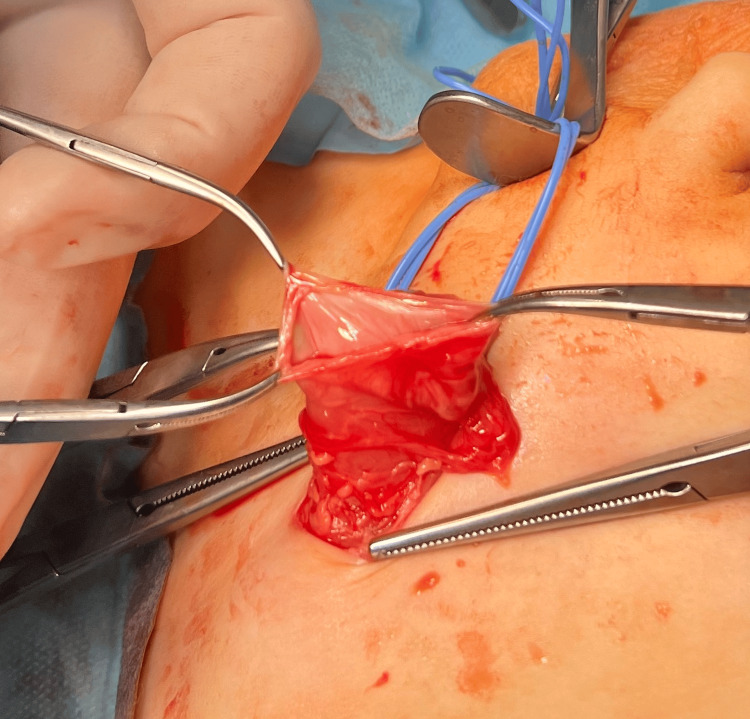
Repair of the recurrent inguinal hernia - first step Classic resection of the hernia sac

**Figure 5:**
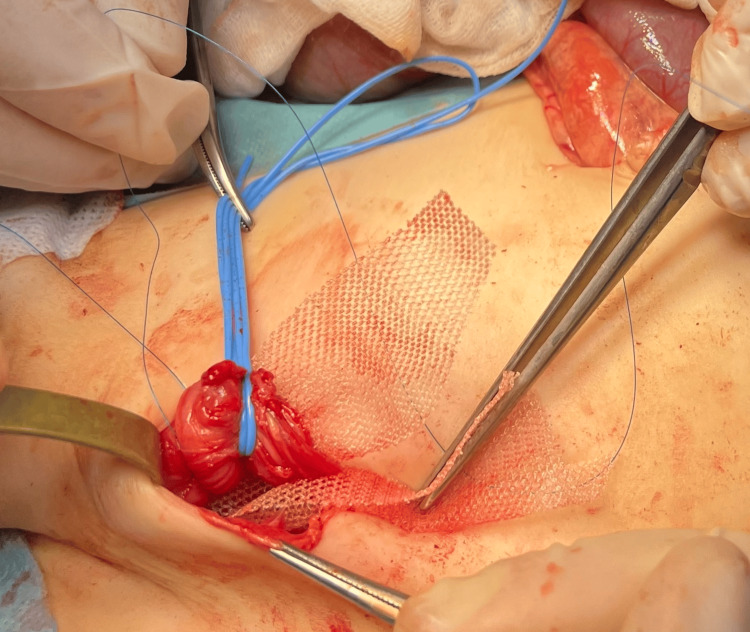
Repair of the recurrent inguinal hernia - second step Lichtenstein technique - implantation of the Vicryl patch

To avoid future recurrences the open revision of the inguinal canal was performed. The hernia was repaired with the classic Lichtenstein technique with a patch implantation, and a resection of the hernia sac (Figures 4, 5).

There were no complications during the postoperative period. The follow-up was eight months, and no hernia recurrence was detected.

## Discussion

Laparoscopic methods for inguinal hernia repair in the pediatric population become an important alternative to open surgeries. Referring to the literature on the PIRS technique the complication rate varies from 2.8% to 5.9% and the recurrence rate varies from 0 to 3% [2,4-6]. Literature provides clinicians with limitations of the PIRS method such as presence of the complicated hydrocele, incarcerated or strangulated hernia, presence of the organ sliding into proximal sac (although it is not always treated as a contradiction to the laparoscopic approach) [1,6,7], but there are no specific studies analyzing whether a comorbidity such as connective tissue disorder should exclude patients from the laparoscopic treatment. Publications describing safety assessments and possible complications in the PIRS method record problems such as recurrence of the hernia, intra- and postoperative bleeding (eg. iliac vessel puncture, inferno epigastric vein injury), stitch abscesses, ecchymosis, persisted postoperative pain, recurrent hydrocele, omental evisceration, keloid scar, swelling of the groin, umbilical incision granuloma, bowel strangulation [1,2,4,5,8-12]. Another problem with the PIRS approach is a possible conversion to an open surgery which can make the procedure significantly longer, but is rarely performed [9]. Many studies prove that the number of complications decreases with the surgeon’s experience and is limited to not-severe problems and low number of recurrences [9,11]. In our case, due to the patient’s many additional health problems, mainly cardiological issues, it was important to choose the simplest and the shortest surgical approach and during one procedure exclude, or repair contralateral hernia, which is feasible in the PIRS method [5,9-15]. Literature provides studies explaining possible reasons for recurrences in laparoscopic repairs of the inguinal hernia such as presence of risk factors causing increased abdominal pressure (eg. cough, constipation, diarrhea), and insufficient surgical training, but analyzed groups of patients are very small and do not describe patients with systemic diseases [16]. To our knowledge there are no studies, or case reports describing the use of the PIRS technique, or other laparoscopic approaches in patients with connective tissue disorders. In our institution the PIRS method was previously used in patients with connective tissue disorders (eg. Marfan syndrome) and no complications, nor recurrences were recorded. In our opinion, the reason for the recurrence in this patient was the low quality of the connective tissue. Moreover, we suspect that, this complication could occur even after the open procedure, where standard operation in the pediatric population involves closing of the patent processus vaginalis without the patch implantation. 

## Conclusions

We conclude that there is insufficient evidence to determine which risk factors are correlated with increased risk of the recurrence in patients with different types of connective tissue disorders. To increase safety, we propose open hernia repairs with the patch implantation to most patients with connective tissue disorders until the risk factors for the recurrence are determined. More research is necessary to determine potential contraindications to the PIRS technique, especially in the context of connective tissue disorders. 
